# Investigation of a food-borne outbreak of gastroenteritis in a school canteen revealed a variant of sapovirus genogroup V not detected by standard PCR, Sollentuna, Sweden, 2016

**DOI:** 10.2807/1560-7917.ES.2017.22.22.30543

**Published:** 2017-06-01

**Authors:** Maria-Pia Hergens, Joanna Nederby Öhd, Erik Alm, Helena H Askling, Sofia Helgesson, Mona Insulander, Nina Lagerqvist, Bo Svenungsson, Malin Tihane, Thomas Tolfvenstam, Per Follin

**Affiliations:** 1 Department of Communicable Disease Control and Prevention, Stockholm County Council, Sweden; 2 Department of Medicine Solna, Infectious Disease Unit, Karolinska Institutet, Stockholm, Sweden; 3 Public Health Agency of Sweden, Stockholm, Sweden

**Keywords:** epidemiology, foodborne infection, gastrointestinal disease, molecular methods, outbreaks, Sapovirus, viral infections, Calicivirus

## Abstract

A food-borne outbreak of gastroenteritis with more than 650 suspected cases occurred in April 2016 in Sollentuna, Sweden. It originated in a school kitchen serving a total of 2,700 meals daily. Initial microbiological testing (for *Campylobacter, Salmonella, Shigella, Yersinia, Giardia, Cryptosporidium, Entamoeba histolytica*, adeno-, astro-, noro-, rota- and sapovirus) of stool samples from 15 symptomatic cases was negative, despite a clinical presentation suggestive of calicivirus. Analyses of the findings from both the Sollentuna municipality environmental team and a web-based questionnaire suggested that the source of the outbreak was the salad buffet served on 20 April, although no specific food item could be identified. Subsequent electron microscopic examination of stool samples followed by whole genome sequencing revealed a variant of sapovirus genogroup V. The virus was not detected using standard PCR screening. This paper describes the epidemiological outbreak investigation and findings leading to the discovery.

## Introduction

Sapovirus causes acute gastroenteritis in humans and belongs to the *Caliciviridae* family, along with norovirus and three other genera [1]. Since the virus was first described in 1976, it has been studied and sapoviruses pathogenic to humans are currently classified into four genogroups [1]. Sapovirus has a worldwide distribution and is a common cause of sporadic gastroenteritis [2-4]. Outbreaks may occur in different settings throughout the year, although the reported outbreaks are fewer than for norovirus. Prevalence is highly variable without any apparent geographical pattern. Prevalence and genotype distribution have shifted over time [5]. Food-borne transmission has been suspected on several occasions [6-8]. A recent summary and analysis of reported food-borne outbreaks in the European Union in 2011 estimated that viruses were the cause in 13% of those outbreaks in which a causative agent was verified or where such outbreaks were associated with sufficient solid data to be categorised as viral outbreaks supported by strong evidence; a majority of these (98%) were caused by the *Caliciviridae* family, specifically noroviruses [9].

On the weekend of 22–24 April 2016, a suspected outbreak of gastroenteritis among students and teachers at four schools in Sollentuna, Sweden, was reported to the municipality of Sollentuna. The initial report stated that more than 50 students and teachers were ill. The Sollentuna municipality environmental team notified the Department of Communicable Disease Control and Prevention in Stockholm on 25 April. All schools in Sollentuna, as well as some outside the municipality, received food from the same central school kitchen, based in one of the affected schools. An outbreak control team was set up to investigate the magnitude of the outbreak and to identify the causative agent of the gastroenteritis in order to localise the source of the outbreak. This report describes the epidemiological investigation and findings, as well as the implemented control measures.

## Methods

The outbreak control team included representatives from the Department of Communicable Disease Control and Prevention in Stockholm and the Sollentuna municipality environmental team. The Public Health Agency of Sweden and the National Food Agency were responsible for the microbiological analyses undertaken during the late phase of the outbreak investigation.

### Descriptive epidemiology

Early on, the investigation revealed that the central kitchen served 2,700 meals per day at 21 schools and preschools, suggesting that a considerably higher proportion of schools in the Stockholm area could have been exposed than was initially thought. The age of students at the 21 schools and preschools ranged from 1 to 15 years.

**Figure 1:**
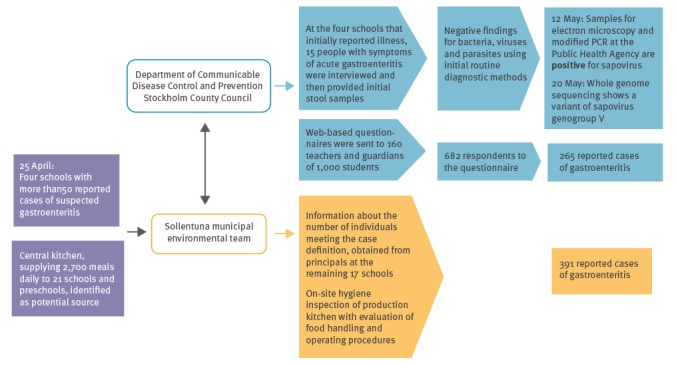
Communication and important moments in the outbreak investigation, food-borne gastroenteritis outbreak, Sollentuna, Sweden, April 2016 (n = 656)

In order to estimate the magnitude of the outbreak, the principals at the school distributed a web-based questionnaire that was provided by the Department of Communicable Disease Control and Prevention in Stockholm to students’ parents, guardians and teachers. Because quick and early distribution was a priority, only the students and teachers at the four schools were included that had initially reported the outbreak to the Sollentuna municipality environmental team. Approximately 1,000 students (6–15 years of age) and 160 teachers attended these four schools, which was considered a sufficient number for meaningful analysis. The questionnaire was distributed by email on the morning of 26 April with a 7-day submission deadline. In addition, to obtain a more complete picture of the magnitude of the outbreak, the Sollentuna municipality environmental team requested that the remaining 17 schools served by the central kitchen estimated the number of students and personnel with gastroenteritis symptoms (Figure 1). Data pertaining to whether the schools received both salad buffet and warm meals were also gathered.

Purple fields: outbreak background information; blue fields: investigation by the Department of Communicable Disease Control and Prevention; yellow fields: investigation by the Sollentuna municipality environmental team. Open arrows: information received by the Sollentuna municipality environmental team; filled arrows: response measures including simultaneously (i) stool sampling for microbial analysis, (ii) distribution of questionnaires and (iii) information gathering as a basis for case finding among individuals who had consumed food prepared in a central school kitchen in Sollentuna municipality, Stockholm, Sweden in April 2016. A total of 656 gastroenteritis cases were reported.

The Sollentuna municipality environmental team was responsible for the environmental investigation, including inspection of the kitchen, review of hygienic procedures and staff hygiene, procedures for safe handling of raw meat, as well as procedures for heating and cooling food. Interviews were conducted with kitchen personnel. Food samples were taken and when possible, attempts were made to trace them back to the source. Stool samples for microbiological analysis were obtained from 15 people with symptoms of gastroenteritis.

#### Case definition and case findings

Initial reports indicated that sporadic cases were reported as early as on 21 April; therefore, we decided to ask about manifestation of symptoms after 20 April. For the purposes of our analytical investigation, a case of gastroenteritis was defined as someone who gave a positive response to the question ‘*Have you had symptoms of gastroenteritis after 20 April’* among students and teachers at any of the four schools that had first notified Sollentuna municipality about the outbreak.

Secondary cases of gastroenteritis were defined as individuals who did not attend any of these four schools, but belonged to a household where a primary case of gastroenteritis was identified as defined above, and who presented with symptoms of gastroenteritis beginning within 72 hours of the primary case.

The case definition of gastroenteritis from the other 17 schools, as reviewed by the Sollentuna municipality environmental team, was based on absences due to gastroenteritis after 22 April self-reported to administrative personnel at these schools.

### Analytical epidemiology

#### Data collection and analysis

The web-based questionnaire included questions about whether the respondents were teachers or students and about their grade level. Also included was information on clinical manifestations, onset and duration of symptoms, as well as questions regarding potential secondary cases. Early telephone interviews with the parents/guardians of 15 of the sick individuals showed onset of symptoms 12–36 hours after the most recent consumption of food at school, high rates of nausea, vomiting and diarrhoea, as well as short (1–2 days) duration of symptoms, which together suggest that the gastroenteritis was probably caused by a virus rather than bacteria, parasites or toxins. The incubation period for the most common viral gastrointestinal illnesses is short, usually 1–2 days [10]. Given the clinical picture and the short incubation period for the most likely viral agents, the form included detailed questions about what food items were consumed at lunch on 20–22 April (assuming a probable incubation period of 1–2 days).

We analysed the distribution of cases by time of onset of symptoms and demographic characteristics, as well as attack rates and unadjusted risk ratios (RR) of gastroenteritis in relation to consumption of each food item. Unadjusted RR were calculated using Episheet [11].

#### Microbiological investigations

Stool samples were collected for analysis from 15 individuals who reported symptoms of gastroenteritis. For logistical reasons, samples were sent to either of two separate clinical microbiological laboratories in Stockholm.

All 15 samples were cultured for *Salmonella*, *Shigella*, *Campylobacter* and *Yersinia*. Because two samples were not available in sufficient quantity, only 13 samples were analysed using the commercially available qPCR geneXpert (Cepheid, Sunnyvale, United States) proprietary platform (adeno-, astro-, noro-, rota- and sapovirus) for seven of the samples and a sapovirus real-time RT-PCR [12] for six of the samples. Eight samples were analysed by microscopy with appropriate staining to search for parasites.

Only six samples were large enough for further analysis by the Public Health Agency of Sweden using electron microscopy, RT-PCR, real-time RT-PCR and whole genome sequencing (WGS). The RT-PCR was performed using the following primers for sapovirus: forward primers CTCGCCACCTACRAWGCBTGGTT, GCCACCTACGAATCCTGGTTCAT, and CAATTGCATGYTACAACAGCTGGTACAT, and reverse primers CGCGCCTCCATRCTACCACCCCA, CGGRCYTCAAAVSTACCNCCCCA, and TGAGACYGTGACTCTRATRTCCATTGC. The real-time RT-PCR was modified from [12]: forward primers GAYCAGGCYCTCGCYACCTAC, TTGGCCCTCGCCACCTAC, and TTTGAACAAGCTGTGGCRTGCTAC, reverse primer NNCCCTCCATYTCAAACACTA and probes FAM-CTGTACCRCCTATGAACCA-MGB and FAM-CTGYACCACCTATRAACCA-MGB.

#### Environmental investigation

Inspectors from the Sollentuna municipality environmental team conducted a post-outbreak inspection of the central kitchen on 25 April. Physical inspection and personnel interviews were carried out to assess seven areas: infrastructure and procedures, equipment and facilities, food products and packaging, safe handling and storage, cleaning procedures, compliance with temperature protocols, as well as personal hygiene and food traceability.

Samples were obtained from saved main courses, as well as from batches of frozen vegetables and herbs served on 20–22 April. Samples were also obtained from batches of salad that were delivered to the central kitchen after the outbreak. No salad from delivery before the outbreak was available. All food samples were initially stored at −26 ^o^C by the Sollentuna municipality environmental team and subsequently sent to the National Food Agency for analysis once results from the human samples became available.

## Results

### Descriptive epidemiology

**Figure 2:**
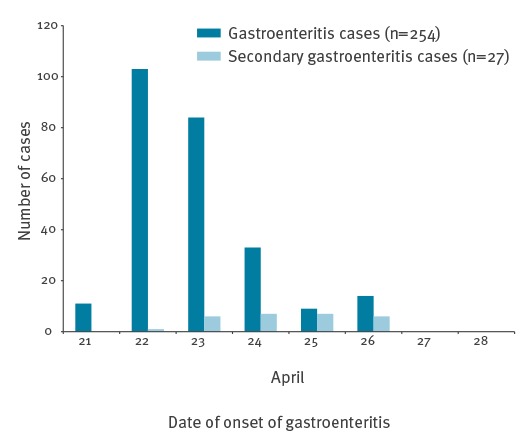
Epidemic curve of onset of gastroenteritis symptoms among students and teachers at four schools and secondary cases, Sollentuna, Sweden, April 2016 (n = 281)

The web-based questionnaire was sent to ca 1,160 people and since all questions were voluntary, the number of responses to each question varied. Of the 682 people (59%) who responded to the question regarding symptoms of gastroenteritis after 20 April, 674 also answered the question concerning sex (363 female and 311 male) and of these, 265 people (39%) reported symptoms of gastroenteritis. Among this latter group, 260 indicated their sex (144 female and 116 male). The attack rate among teachers was 54% (65 of 121), while in students it was 36% (198 of 553). For eight people, information regarding teacher/student status was lacking. The attack rate did not differ significantly between men and women, with 37% (116/311) and 40% (144/360), respectively. Information regarding sex was missing for 11 people. Among all self-reported cases of gastroenteritis, only 10 respondents indicated that they had not eaten in the canteen. Figure 2 shows the distribution of cases by date of onset of gastroenteritis symptoms; cases started to occur on 21 April, followed by a peak on 22 April. Among these cases, 10% (27/281) were reported as secondary cases.

The summary of the investigation conducted by the Sollentuna municipality environmental team on 25 April showed that 123 teachers and 268 students from the 17 schools not included in the initial investigation had reported symptoms of gastroenteritis (see Table 1). With the addition of these 391 cases, more than 650 people reportedly had gastroenteritis symptoms associated with the outbreak overall.

**Table 1:** Students/children and teachers reporting symptoms of gastroenteritis after consuming food provided by a central school kitchen to 17 preschools and schools, Sollentuna, Sweden April 2016 (n = 391)

	Received warm meals	Received salad buffet	Teachers with GE symtoms^a^	Students/children with GE symptoms^a^
School/preschool 1	Yes	No	0	0
School/preschool 2	Yes	Yes	8	1
School/preschool 3	Yes	Yes	5	15
School/preschool 4	Yes	No	0	0
School/preschool 5	Yes	Yes	3	NA
School/preschool 6	Yes	Yes	7	5
School/preschool 7	Yes	Yes	6	21
School/preschool 8	Yes	Yes	1	1
School/preschool 9	Yes	No	0	0
School/preschool 10	Yes	Yes	3	4
School/preschool 11	Yes	Yes	7	6
School/preschool 12	Yes	Yes	6	5
School/preschool 13	Yes	Yes	10	17
School/preschool 14	Yes	Yes	9	10
School/preschool 15	Yes	Yes	21	78
School/preschool 16	Yes	Yes	8	32
School/preschool 17	Yes	Yes	29	73

GE: gastroenteritis; NA: not available.

^a^ Self-reported cases according to the school principals; these cases were not included in further analytical investigation since the individuals involved did not receive the web-based questionnaire.

**Figure 3:**
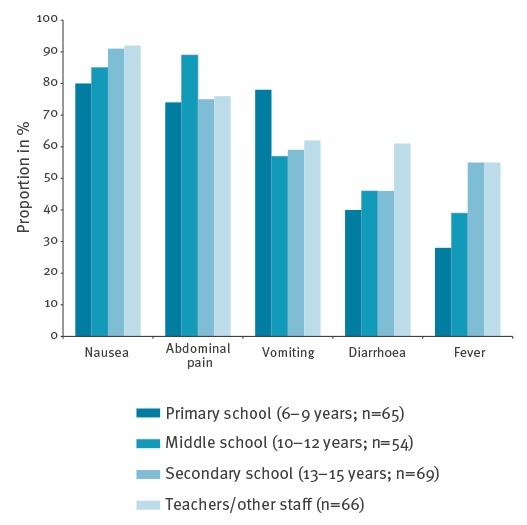
Proportion of predominant gastroenteritis symptoms in different age groups among students and teachers at four schools, Sollentuna, Sweden, April 2016 (n = 254)

According to the questionnaires, the most prevalent clinical symptoms were nausea (246/265; 93%), abdominal pain (220/265; 83%), vomiting (183/265; 69%), diarrhoea (133/265; 50%) and fever (127/265; 48%). Duration of these symptoms varied somewhat in that vomiting was of shorter duration than diarrhoea (data not shown). Stratifying symptoms by age (6–9 years: n = 65; 10–12 years: n = 54; 13–15 years: n = 69; teachers: n = 66) showed that nausea, diarrhoea and especially fever were more commonly reported by the older age groups (Figure 3). To our knowledge none of the reported cases required hospital admission.

The figure does not include secondary cases.

### Analytical epidemiology

The Sollentuna municipality environmental team found (Table 1) that cases of gastroenteritis were reported only from the 14 schools and preschools that received both the main course and the salad buffet, whereas no cases of gastroenteritis were reported from the three schools that did not receive the salad buffet. Analysis of the questionnaires found no specific food item to be associated with confirmed case status. However, consumption of mixed salad, mixed beans or green beans at lunch on 20 April was associated with confirmed case status, with RR of 2.0 (95% confidence interval (CI): 1.6–2.6), 2.1 (95% CI: 1.6–2.6) and 2.0 (95% CI: 1.6–2.6), respectively (Table 2). Food items served on 21 April or 22 April did not show RR above 1.8. Analyses of the findings from both the Sollentuna municipality environmental team and the web-based questionnaire suggested that the source of the outbreak was the salad buffet served on 20 April, although no specific food item could be identified.

**Table 2:** Attack rate and crude risk ratios for gastroenteritis among students and teachers at four schools, by food item served in the canteen between Wednesday, 20 April and Friday 22 April 2016, Sollentuna, Sweden (n = 265)

	Food item	Attack rate						Risk ratio
		Among exposed			Among unexposed			
		Cases	Total	%	Cases	Total	%	RR (95% CI)
Wednesday 20 April	Pasta	174	387	45	10	37	27	1.7 (1.0–2.9)
	Minced meat sauce	157	347	45	20	66	30	1.5 (1.0–2.2)
	Vegetarian sauce	13	31	42	122	283	43	1.0 (0.6–1.5)
	Mixed salad	48	71	68	76	226	34	2.0 (1.6–2.6)
	Green lettuce	53	107	50	81	215	38	1.3 (1.0–1.7)
	Tomatoes	77	140	55	58	184	32	1.7 (1.3–2.3)
	Cucumber	112	231	48	38	112	34	1.4 (1.0–1-9)
	Mixed beans	41	56	73	91	255	36	2.1 (1.6–2.6)
	Carrots	64	129	50	72	188	38	1.3 (1.0–1.7)
	Haricots verts	37	52	71	89	252	35	2.0 (1.6–2.6)
Thursday 21 April	Chicken curry	147	323	46	29	84	35	1.3 (1.0–1.8)
	Samosas	17	44	39	116	263	44	0.9 (0.6–1.3)
	Rice	151	343	44	27	64	42	1.0 (0.8–1.4)
	Garlic sauce	23	55	42	106	247	43	1.0 (0.7–1.4)
	Green lettuce	58	114	51	85	221	38	1.3 (1.0–1.7)
	Tomatoes	68	130	52	70	195	36	1.5 (1.1–1.9)
	Carrots	45	92	49	90	227	40	1.2 (0.9–1.6)
	Cucumber	96	212	45	53	128	41	1.1 (0.8–1.4)
	Mixed vegetables	27	38	71	108	275	39	1.8 (1.4–2.3)
	Peas	37	70	53	100	249	40	1.3 (1.0–1.7)
	Pears	24	56	43	106	251	42	1.0 (0.7–1.4)
	Mushrooms	31	51	61	107	273	39	1.6 (1.2–2.0)
Friday 22 April	Broccoli soup	83	163	51	91	219	42	1.2 (1.0–1.5)
	Pancakes	156	379	41	34	68	50	0.8 (0.6–1.1)
	Strawberry jam	138	342	40	45	90	50	0.8 (0.6–1.0)
	Green lettuce	32	66	48	120	279	43	1.1 (0.8–1.5)
	Tomatoes	46	86	53	101	255	40	1.4 (1.1–1.7)
	Carrots	60	130	46	93	218	43	1.1 (0.9–1.4)
	Cucumber	69	151	46	85	203	42	1.1 (0.9–1.4)
	Cauliflower	16	29	55	129	301	43	1.3 (0.9–1.8)
	Baby corn	29	70	41	117	266	44	0.9 (0.7–1.3)
	Mushrooms	27	45	60	122	294	41	1.4 (1.1–1.9)

CI: confidence interval; RR: risk ratio.

### Laboratory investigation

The 15 stool samples were negative for *Salmonella*, *Shigella* and *Campylobacter*. Further analyses on 13 of these 15 samples also returned negative for adeno-, astro-, noro-, rota- and sapovirus. Material in the remaining two samples was not sufficient for virus analysis. All samples were negative for parasites (*Giradia*, *Cryptosporiduim* and *Entamoeba histolytica*). Two samples (sibling cases) were positive for *Yersiniaenterocolitica* 1A.

**Figure 4:**
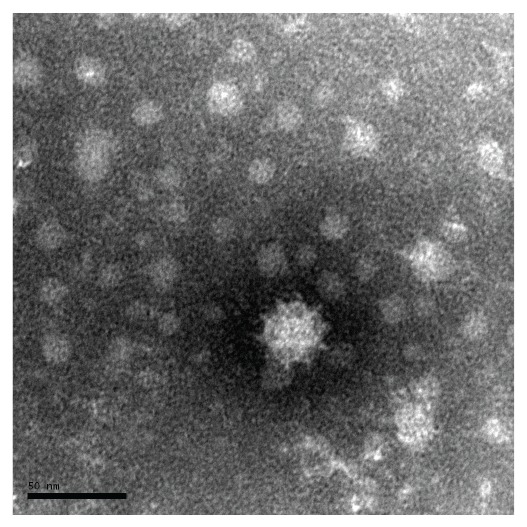
Electron micrograph of negatively stained sapovirus, gastroenteritis outbreak, Sollentuna, Sweden, April 2016

Subsequently, six samples had enough material left to be sent for further analysis to the Public Health Agency of Sweden, which found calicivirus in three of the samples using electron microscopy (Figure 4). All samples were also analysed using RT-PCR and WGS, in which a variant of sapovirus genogroup V was found in five of six samples.

Scale: bar indicates 50 nm.

### Environmental investigation

Among kitchen staff, three of 11 had symptoms of gastroenteritis during the outbreak. Two of the three had eaten the same food as the students. Stool samples collected from the three symptomatic kitchen workers were found to be negative in routine analysis. No specimens from these kitchen workers were among those sent to the Public Health Agency of Sweden.

#### Post-outbreak measures taken by the central kitchen

At the request of the Sollentuna municipality environmental team, stored frozen food was discarded and the facility, salad bar, cutting boards and utensils were thoroughly cleaned and disinfected.

#### Analysis of food samples

The central kitchen had been cleaned on the Friday preceding the outbreak and many leftover food items had already been discarded. Therefore, collection of food samples was highly limited or from food unrelated to the outbreak. Frozen parsley had been included in several dishes served on 20 April and sufficient amounts of parsley and green beans remained for analysis.

After results from the human stool samples became available from the Public Health Agency of Sweden, parsley and green beans were sent to the National Food Agency for RT-PCR testing with appropriate primers for sapovirus. Both these food sources were negative. Mixed salad or mixed beans from 20 April were not available.

## Discussion

To our knowledge, this food-borne outbreak represents one of the major sapovirus outbreaks since the one in Japan in 2010 [7]. Overall, the Sollentuna outbreak in 2016 involved more than 650 reported cases of gastroenteritis in both children and adults. Sapovirus is known to cause viral gastroenteritis in young children, whereas adults seem to be less affected [2-4]. However, in this outbreak, older students and teachers appeared to be equally or even more affected than young children. The attack rate among teachers was higher than in students. This difference may be due to selection bias as the response rate was 75% among teachers and only 35% among students. This would be true if guardians of asymptomatic students were more likely to respond to the questionnaire, but this seems unlikely.

Assuming that all 2,700 meals served were consumed, causing ca 650 primary cases, this could indicate an attack rate of at least 24%. The most commonly reported symptoms were nausea and abdominal pain, followed by vomiting and diarrhoea. Fever correlated with increasing age in accordance with previous results [13]. Other sapovirus outbreaks have reported fever as the most common symptom and diarrhoea as the least common [13]. Studies comparing genogroup-specific differences and prevalence of various symptoms have found no significant associations to explain the varying clinical presentations in different outbreaks, the reason for which remains unclear [14].

Two siblings were initially found to be positive in stool cultures for *Y.enterocolitica* 1A. However, there is controversy regarding the pathogenicity of *Y.enterocolitica* 1A [15] and considering that it was only found in two siblings and no other students, this could not explain the outbreak and was hence considered to be an incidental finding.

No clear source of the outbreak was identified, although food items served in the salad buffet were suspected. The web-based questionnaire showed that mixed salad, mixed beans and green beans all had case-related RR of ca 2.0. Also, the findings of the Sollentuna municipality environmental team implicated the salad buffet (Table 1). Vegetable components including mixed salad and frozen vegetables have been reported in several food-borne outbreaks caused by caliciviruses, more specifically norovirus. Food-borne outbreaks caused by sapovirus are less common [9]. Lack of adequate food samples made it impossible to verify the results by analysing food items. Some studies show that asymptomatic food handlers have high viral loads of sapovirus [6,13,16] and may pose potential risk of secondary transmission. Of 11 kitchen workers, three reported symptoms during the outbreak which coincided with the peak of the outbreak. The questionnaire was available for seven days and as shown in the epidemic curve, symptom onset of some cases occurred almost one week after assumed exposure, which may suggest that these could in fact be secondary cases. However, we have no reason to believe that such secondary cases could have had much impact on the results.

## Conclusion

The rapid and efficient multidisciplinary collaboration made it possible to estimate the magnitude of the outbreak, present a descriptive epicurve, provide information to the affected schools, suggest precautions and identify a plausible aetiology (calicivirus) within a few days. Despite the operational efficiency, epidemiological and microbiological evidence remained elusive, while the electron microscopy findings with whole genome sequencing represent a crucial breakthrough. Whole-genome sequencing revealed a sapovirus variant clustering with genogroup V. Phylogenetic analysis of the capsid gene showed that the sequences of S3 and S6 clustered with sapovirus genogroup V but clearly separated from almost all other isolates in the genogroup [17].The investigation of this outbreak clearly demonstrates the importance of epidemiological analysis coupled with both conventional and new microbiological techniques, especially when searching for new variants of infectious agents.
